# Long-term use of anti-cholesterol drugs and cancer risks in a Japanese population

**DOI:** 10.1038/s41598-024-53252-4

**Published:** 2024-02-05

**Authors:** Yuki Okita, Tomotaka Sobue, Ling Zha, Tetsuhisa Kitamura, Motoki Iwasaki, Manami Inoue, Taiki Yamaji, Shoichiro Tsugane, Norie Sawada

**Affiliations:** 1 Division of Environmental Medicine and Population Sciences, Department of Social Medicine, Graduate School of Medicine, Osaka University, Suita, Osaka 565-0871 Japan; 2 Division of Epidemiology, National Cancer Center Institute for Cancer Control, Chuo-Ku, Tokyo 104-0045 Japan; 3 Division of Cohort Research, National Cancer Center Institute for Cancer Control, Chuo-Ku, Tokyo 104-0045 Japan; 4 Division of Prevention, National Cancer Center Institute for Cancer Control, Chuo-Ku, Tokyo 104-0045 Japan; 5 National Institute of Health and Nutrition, National Institutes of Biomedical Innovation, Health and Nutrition, Shinjuku-Ku, Tokyo 162-8636 Japan

**Keywords:** Cancer, Risk factors

## Abstract

Several studies have investigated the association between the use of anti-cholesterol drugs and cancer risks, of which results have been inconsistent. This study included 67,768 participants from the Japan Public Health Center-based Prospective Study. The data on anti-cholesterol drug use was collected using three questionnaires of the survey conducted every five years. We divided the participants into three groups according to the duration of the anti-cholesterol drug use. Multivariable-adjusted Cox proportional hazard regression models were used to calculate hazard ratios (HR) and 95% confidence intervals (CI). During the 893,009 person-years of follow-up from the 10-year follow-up survey, 8,775 participants (5,387 men and 3,388 women) were newly diagnosed with cancers. The duration of anti-cholesterol drug use was significantly associated with a decreased risk of liver cancer (HR:0.26, 95% CI 0.11–0.64 in > 5 y group) and with an increased risk of pancreatic cancer (HR:1.59, 95% CI 1.03–2.47 in > 5 y group). Moreover, a different trend was observed between men and women in the association with the risk of lung cancer. This study suggested that long-term use of anti-cholesterol drugs may have associations with a decreased incidence of liver cancer and with an increased incidence of pancreatic cancers.

## Introduction

The most commonly used anti-cholesterol drugs currently are statins, otherwise known as 3-hydroxy-3-methylglutaryl-coenzyme A (HMG-CoA) reductase inhibitors, and statins have been proven to reduce the risk of cardiovascular diseases ^1^. Moreover, statins are among the most widely used drugs to prevent the development of cardiovascular diseases ^2^. Furthermore, statins show various characteristics independently from their lipid-lowering effects ^3^. It has been suggested in preclinical and clinical studies as evidence that statins have effects of the inhibition of tumor growth and the induction of apoptosis in specific cancer cell types ^4,5^. The effects of statins on cerebrovascular and cardiovascular diseases are well known. In contrast, the effects of statins on risk of cancer have not been clarified fully yet and available findings have been inconsistent.

A meta-analysis of data from 26 randomized controlled studies found no association between the use of statin and cancer incidence ^6^. However, the periods of intervention and follow-up were relatively short (2 to 10.4 years) and only two of these studies had follow-up of over 5.2 years including the period of intervention. Observational studies to evaluate the association between long-term use (typically not more than 4 or 5 years) and cancer risks did not show strong or consistent associations between statin use and cancer incidence in total or for any specific cancers ^7^. One of the most comprehensive studies to investigate the association between long-term statin use and cancer risks examined the risk of more than 25 types of cancer ^8^. This study provided no strong evidence of either causation or prevention of any cancer by statin. However, this study had a limitation of the absence of information on potential confounders, including smoking.

Therefore, we conducted a large-scale population-based cohort study in Japan to investigate the association between long-term use of anti-cholesterol drugs, mainly statins, and the risk of various cancers.

## Materials and methods

### Study population

The Japan Public Health Center-based Prospective Study (JPHC study) is a large population-based cohort study that assessed the risk factors for cancer, metabolic diseases, and other lifestyle-related diseases. This study consisted of two cohorts, and the participants were Japanese residents registered in 11 public health center (PHC) areas. Details of the study design are available elsewhere ^9^.

**Figure 1:**
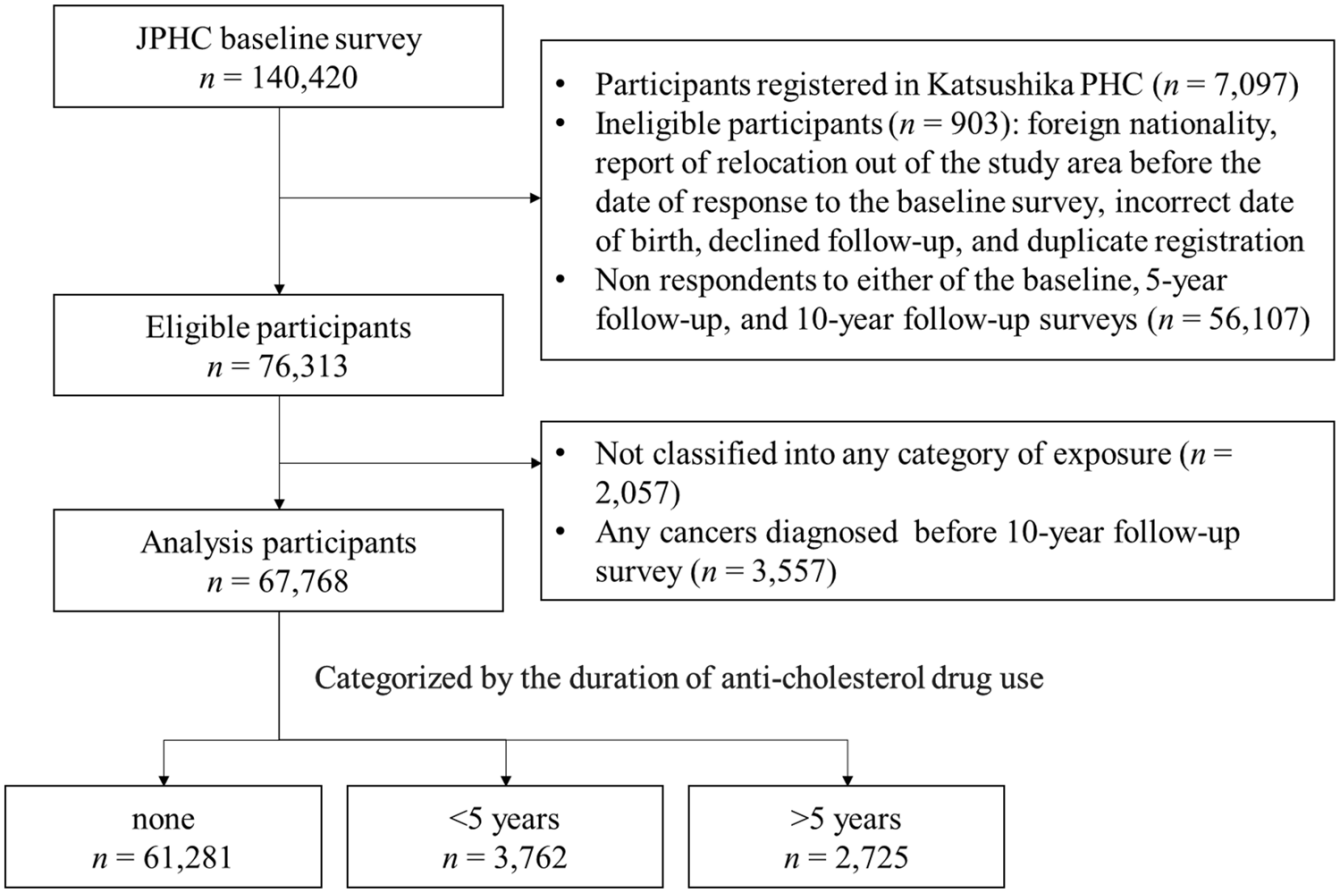
Flowchart of study participants. The JPHC study enrolled 140,420 participants at the baseline. The exclusion criteria were applied and the final cohort for analysis consisted of 67,768 participants. Participants were divided into the following four categories (none, < 5 years, 5–10 years, and > 10 years) according to the duration of anti-cholesterol drug use up to the start of the follow-up period at which the 10-year follow-up survey was conducted. JPHC study, Japan Public Health Center-based Prospective Study.

Cohort I and Cohort II together enrolled 140,420 participants between 1990–1994 and 1993–1995, respectively. Figure 1 shows the flowchart of participants in this study. Participants in the Katsushika PHC area (n = 7,097) were excluded because different definitions for the study population were applied. Additionally, 903 participants were excluded with the following reasons: foreign nationality, report of relocation out of the study area before the date of response to the baseline survey, incorrect date of birth, declined follow-up, and duplicate registration. Participants who responded to all the baseline, 5-year follow-up, and 10-year follow-up surveys were included (n = 76,313). Each individual answered the questionnaire asking whether they were currently taking anti-cholesterol drugs or not at all the surveys and classified into the categories defined in the section of “Exposure assessment”. Participants who stopped or resumed between the baseline and 10-year follow-up were also excluded because they were not classified into any defined categories of exposure (n = 2,057). Furthermore, we also excluded participants who got diagnosed with cancer before the 10-year follow-up survey (n = 3,557), died, moved out of the study area, or those who were lost to follow-up before the 10-year follow-up survey (n = 2,931). This is because the follow-up period started at the 10-year follow-up survey. Therefore, the final cohort for analysis consisted of 67,768 participants in this study.

### Exposure assessment

Participants were divided into the following four categories at first according to the duration of anti-cholesterol drug use up to the start of the follow-up period at which the 10-year follow-up survey was conducted (none, < 5 years, 5–10 years, and > 10 years). The period of anti-cholesterol drug use was based on the baseline, 5-year follow-up, and 10-year follow-up questionnaires asking whether they were currently taking anti-cholesterol drugs or not. Because the population size in the categories of “5–10 years” and “ > 10 years” is too small to evaluate the association appropriately, those two categories were integrated into one category which is defined as “ > 5 years” and the final categorization used for the analysis were “none”, “ < 5 years”, and “ > 5 years”.

The category of “none” included participants who did not take anti-cholesterol drugs at baseline, 5-year follow-up, or 10-year follow-up (n = 61,281). The category of “ < 5 years” included participants who took anti-cholesterol drugs at the 10-year follow-up and not at baseline, or from the 5-year follow-up (n = 3,762). The category of “ > 5 years” included participants (n = 2,725) who took anti-cholesterol drugs both at the 5-year and 10-year follow-up but not at baseline (“5–10 years”, n = 2,028) and who took anti-cholesterol drugs at baseline, 5-year follow-up, and 10-year follow-up (“ > 10 years”, n = 697).

### Follow-up and case identification

The follow-up of the participants was started at the 10-year follow-up survey through December 31, 2013, using residential status and survival status which was collected through the residential registers of each study area or the municipal office of areas to which they moved out. The diagnosis of cancers was captured during the follow-up period the 10-year follow-up survey. We selected the most common cancers in Japan based on national cancer registration. Our analyses included esophageal, stomach, colorectal, liver, biliary tract, pancreatic, lung, breast, uterine, prostate, and renal cancers ^10^. Cancers were identified using the codes of the International Classification of Diseases for Oncology, third edition ^11^: esophageal cancer = C15, stomach cancer = C16, colorectal cancer = C18, C19, and C20, liver cancer = C22, biliary tract cancer = C23 and C24, pancreatic cancer = C25, lung cancer = C34, breast cancer = C50, uterine cancer = C53, prostate cancer = C61, and renal cancer = C64. For participants diagnosed with more than one cancer during the follow-up periods, the first diagnosed cancer was used for the analysis.

### Statistical analysis

Person-years of follow-up for each participant were calculated from the date of the 10-year follow-up survey to whichever of these endpoints occurred first: diagnosis of any cancer, relocation outside the study area, death, loss to follow-up, or the end of the study period (December 31, 2013).

The survey consisted of questions including various lifestyle risk factors and medical history. Weekly alcohol consumption was calculated using the baseline questionnaire on alcohol consumption which was related to the frequency and the average daily volume of consumption and types of beverages ^12,13^. The cumulative exposure to smoking was assessed in pack-years (PY) using the daily number of cigarettes and the number of years of smoking among former and current smokers. The categories of PY were 0, 1–19, 20–29, 30–39, or ≥ 40.

Cox proportional hazard regression models were used to calculate the HRs, 95% CIs, and *P* trends for all cancers and each cancer type among the four anti-cholesterol drug use categories. Multivariable-adjusted model was adjusted with the following potential confounders identified from the baseline survey: age (continuous), sex, study area (10 PHC areas), body mass index (14 to < 18.5 kg/m^2^, 18.5 to < 25 kg/m^2^, 25 to < 30 kg/m^2^, or 30 to < 40 kg/m^2^), ethanol intake (never, occasional, < 150 g/week, 150 to < 300 g/week, 300 to < 450 g/week, ≥ 450 g/week, or unknown), cumulative exposure to cigarette smoking (PY category), coffee intake (none, 1–3 cups/week, 1–2 cups/day, ≥ 3 cups/day, or unknown), physical activity (none, 1–3 times/month, ≥ 1 times/week, or unknown), occupation (full-time agriculture/forestry/fishery, full-time salaried/self-employed/professional, multiple occupations, full-time housework/retired/unemployed, or unknown), family history of cancer (yes or no), history of diabetes (no, yes, or unknown), and history of hypertension (no, yes, or unknown).

The factors used for the analysis of each specific cancer type were as follows. History of chronic hepatitis or cirrhosis (no, yes, or unknown) for liver cancer. Menopausal status (no, yes, or unknown) and use of hormonal agents (no, yes, or unknown) for breast and uterine cancers.

All statistical analyses were conducted using Stata version 17.0 (StataCorp LP, College Station, TX, USA). All reported *p*-values were two-sided, with a *p-value* of < 0.05 as the statistically significant level.

### Ethics approval and consent to participate

The study was conducted in compliance with the provisions of the Declaration of Helsinki. The study protocol was approved by the institutional review board of the National Cancer Center, Tokyo, Japan (13-021), and by the ethical review board of Osaka University, Osaka, Japan (14020). The participants were informed of the study objectives, and those who completed the survey questionnaire were regarded as having consented to participation. This study was launched before the enactment of ethical guidelines in Japan, and thus obtaining written informed consent was not mandatory. In accordance with the ethical guidelines enacted subsequent to the launch of this study, a research summary was published on the homepage which guarantees participants the opportunity to refuse participation (https://epi.ncc.go.jp/jphc/764/3701.html).

## Results

During the 893,009 person-years of follow-up, 8,775 participants (5,387 men and 3,388 women) were documented as cancer incident cases. The baseline characteristics of participants in the three categories of anti-cholesterol drug use are shown in Table 1. The participants taking anti-cholesterol drugs longer tended to be older. The average age of the men and women in the category of > 5 years was 2.1 and 4.5 years older than that in the “none” category, respectively. The proportion of participants with a history of hypertension and the proportion of female participants in menopause tended to increase as the duration of anti-cholesterol drug use increased.

**Table 1:** Participant characteristics according to the anti-cholesterol drug use categories.

	None	< 5 years	> 5 years	*P-value*
*Men (n* = *30,406)*	28,558	1,143	705	
Age (years, mean) ± SD	51.3 ± 7.7	53.3 ± 7.5	53.4 ± 7.5	< 0.001
BMI (kg/m^2^), %				< 0.001
14– < 18.5	694 (2.4)	11 (1.0)	4 (0.6)	
18.5– < 25	20,014 (70.1)	707 (61.9)	426 (60.4)	
25– < 30	7,053 (24.7)	387 (33.9)	245 (34.8)	
30– < 40	516 (1.8)	29 (2.5)	23 (3.3)	
Missing	281 (1.0)	9 (0.8)	7 (1.0)	
Alcohol drinking, %				0.019
Never	5,914 (20.7)	265 (23.2)	183 (26.0)	
Occasional	2,632 (9.2)	104 (9.1)	55 (7.8)	
< 150 (ethanol g/wk)	6,621 (23.2)	261 (22.8)	159 (22.6)	
150 ≤ , < 300 (ethanol g/wk)	5,837 (20.4)	240 (21.0)	132 (18.7)	
300 ≤ , < 450 (ethanol g/wk)	3,774 (13.2)	138 (12.1)	88 (12.5)	
450- (ethanol g/wk)	3,167 (11.1)	124 (10.8)	76 (10.8)	
Unknown	613 (2.1)	11 (1.0)	12 (1.7)	
Smoking, %				< 0.001
Never	7,174 (25.1)	278 (24.3)	172 (24.4)	
Former	6,657 (23.5)	299 (26.2)	243 (34.5)	
Current	14,549 (50.9)	560 (49.0)	287 (40.7)	
Unknown	138 (0.5)	6 (0.5)	3 (0.4)	
Coffee intake, %				0.055
None	8,517 (29.8)	379 (33.2)	226 (32.1)	
1–3 cup/wk	8,724 (30.5)	314 (27.5)	198 (28.1)	
1–2 cup/day	7,246 (25.4)	303 (26.5)	189 (26.8)	
≥ 3 cup/day	3,792 (13.3)	138 (12.1)	82 (11.6)	
Unknown	279 (1.0)	9 (0.8)	10 (1.4)	
Physical activity, %				0.79
None	18,253 (63.9)	726 (63.5)	438 (62.1)	
1–3/month	4,696 (16.4)	183 (16.0)	118 (16.7)	
≥ 1/wk	5,320 (18.6)	226 (19.8)	141 (20.0)	
Unknown	289 (1.0)	8 (0.7)	8 (1.1)	
Occupation, %				< 0.001
Agriculture/forestry/fishery	6,435(22.5)	222 (19.4)	115 (16.3)	
Salaried/self-employed/professional	18,762 (65.7)	745 (65.2)	467 (66.2)	
Multiple occupations	1,268 (4.4)	55 (4.8)	29 (4.1)	
Housework/retired/unemployed	1,409 (4.9)	92 (8.0)	70 (9.9)	
Unknown	684 (2.4)	29 (2.5)	24 (3.4)	
Family history of cancer, %	22,402 (78.4)	883 (77.3)	526 (74.6)	0.034
History of DM, %	1,549 (5.4)	105 (9.2)	55 (7.8)	< 0.001
History of hypertension, %	4,401 (15.4)	347 (30.4)	164 (37.4)	< 0.001
History of hepatitis / cirrhosis, %	509 (1.8)	22 (1.9)	24 (3.4)	< 0.001
***Women (n* = *37,362)***	32,723	2,619	2,020	
Age (years, mean) ± SD	51.5 ± 7.8	53.8 ± 7.3	56.0 ± 6.6	< 0.001
BMI (kg/m2), %				< 0.001
14- < 18.5	1,121 (3.4)	68 (2.6)	39 (1.9)	
18.5- < 25	22,730 (69.5)	1,666 (63.6)	1,185 (58.7)	
25- < 30	7,629 (23.3)	764 (29.2)	697 (34.5)	
30- < 40	903 (2.8)	91 (3.5)	83 (4.1)	
Missing	340 (1.0)	30 (1.1)	16 (0.8)	
Alcohol drinking, %				0.043
Never	25,279 (77.3)	2,080 (79.4)	1,621 (80.2)	
Occasional	3,337 (10.2)	243 (9.3)	170 (8.4)	
< 150 (ethanol g/wk)	3,146 (9.6)	230 (8.8)	185 (9.2)	
150 ≤ , < 300 (ethanol g/wk)	434 (1.3)	32 (1.2)	25 (1.2)	
300 ≤ , < 450 (ethanol g/wk)	116 (0.4)	6 (0.2)	5 (0.2)	
450- (ethanol g/wk)	110 (0.3)	5 (0.2)	4 (0.2)	
Unknown	301 (0.9)	23 (0.9)	10 (0.5)	
Smoking, %				0.024
Never	30.429 (93.0)	2,449 (93.5)	1,901 (94.1)	
Former	467 (1.4)	39 (1.5)	37 (1.8)	
Current	1,663 (5.1)	114 (4.4)	77 (3.8)	
Unknown	164 (0.5)	17 (0.6)	5 (0.2)	
Coffee intake, %				< 0.001
None	10,239 (31.3)	914 (34.9)	847 (41.9)	
1–3 cup/wk	9,688 (29.6)	831 (31.7)	591 (29.8)	
1–2 cup/day	9,373 (28.6)	680 (26.0)	477 (23.6)	
≥ 3 cup/day	3.095 (9.5)	167 (6.4)	91 (4.5)	
Unknown	328 (1.0)	27 (1.0)	14 (0.7)	
Physical activity, %				< 0.001
None	24,221 (74.0)	1,918 (73.2)	1,459 (72.2)	
1–3 /month	2,459 (7.5)	167 (6.4)	104 (5.1)	
≥ 1 /wk	5,612 (17.2)	488 (18.6)	435 (21.5)	
Unknown	431 (1.3)	46 (1.8)	22 (1.1)	
Occupation, %				< 0.001
Agriculture/forestry/fishery	6,261 (19.1)	443 (16.9)	322 (15.9)	
Salaried/self-employed/professional	12,326 (37.7)	915 (34.9)	605 (30.0)	
Multiple occupations	2,057 (6.3)	166 (6.3)	140 (6.9)	
Housework/retired/unemployed	11,248 (34.4)	1,048 (40.0)	915 (45.3)	
Unknown	831 (2.5)	47 (1.8)	38 (1.9)	
Family history of cancer, %	25,674 (78.5)	2,005 (76.6)	1,513 (74.9)	< 0.001
History of DM, %	751 (2.3)	108 (4.1)	109 (5.4)	< 0.001
History of hypertension, %	4,380 (13.4)	647 (24.7)	702 (34.8)	< 0.001
History of hepatitis / cirrhosis, %	252 (0.8)	13 (0.5)	10 (0.5)	< 0.001
Menopause, %	17,994 (59.8)	1,748 (67.1)	1,611 (79.8)	< 0.001
Use of hormonal drug, %	359 (1.1)	31 (1.2)	22 (1.1)	0.002

The HRs and 95% CIs of cancer incidence by the categories of the anti-cholesterol drug use in the total population are shown in Table 2. The duration of anti-cholesterol drug use showed significant association with a decreased risk of liver cancer (HR:0.26, 95% CI:0.11–0.64 in > 5 years group; *P* for trend = 0.001), and with an increased risk of pancreatic cancer (HR:1.59, 95% CI:1.03–2.47 in > 5 years group). For the other cancers, significant association was not observed between risk of cancer and the long-term use of anti-cholesterol drugs.

**Table 2:** Hazard ratios for major cancer types according to anti-cholesterol drug use categories.

	**None**	** < 5 years**	** > 5 years**	***P* for trend**
**All cancers**				
Cases (n = 8,775)	7,947	459	369	
Univariable HRs (95% CI)	Ref	0.94 (0.86–1.03)	1.05 (0.95–1.17)	0.816
Multivariable HRs (95% CI)	Ref	0.94 (0.86–1.04)	1.03 (0.92–1.14)	0.865
**Esophageal cancer**				
Cases (n = 254)	239	9	6	
Univariable HRs (95% CI)	Ref	0.61 (0.31–1.19)	0.57 (0.25–1.28)	0.062
Multivariable HRs (95% CI)	Ref	0.85 (0.43–1.66)	0.88 (0.39–2.01)	0.623
**Stomach cancer**				
Cases (n = 1,461)	1,330	58	55	
Univariable HRs (95% CI)	Ref	0.71 (0.55–0.92)	1.25 (0.99–1.58)	0.703
Multivariable HRs (95% CI)	Ref	0.73 (0.56–0.95)	1.22 (0.96–1.56)	0.706
**Colorectal cancer**				
Cases (n = 1,644)	1,476	102	66	
Univariable HRs (95% CI)	Ref	1.12 (0.92–1.37)	1.02 (0.79–1.30)	0.519
Multivariable HRs (95% CI)	Ref	1.09 (0.89–1.34)	0.94 (0.73–1.20)	0.958
**Liver cancer**				
Cases (n = 403)	385	13	5	
Univariable HRs (95% CI)	Ref	0.55 (0.32–0.95)	0.29 (0.12–0.71)	0.001
Multivariable HRs (95% CI)	Ref	0.58 (0.33–1.02)	0.26 (0.11–0.64)	< 0.001
**Biliary tract cancer**				
Cases (n = 254)	220	19	15	
Univariable HRs (95% CI)	Ref	1.41 (0.88–2.25)	1.55 (0.92–2.61)	0.039
Multivariable HRs (95% CI)	Ref	1.17 (0.73–1.89)	1.10 (0.64–1.88)	0.565
**Pancreatic cancer**				
Cases (n = 335)	296	16	23	
Univariable HRs (95% CI)	Ref	0.88 (0.53–1.45)	1.77 (1.16–2.70)	0.040
Multivariable HRs (95% CI)	Ref	0.84 (0.50–1.39)	1.59 (1.03–2.47)	0.132
**Lung cancer**				
Cases (n = 1,073)	978	52	43	
Univariable HRs (95% CI)	Ref	0.86 (0.65–1.14)	1.00 (0.73–1.35)	0.614
Multivariable HRs (95% CI)	Ref	0.94 (0.71–1.25)	1.13 (0.83–1.55)	0.637
**Breast cancer**				
Cases (n = 496)	442	29	25	
Univariable HRs (95% CI)	Ref	1.07 (0.73–1.55)	1.28 (0.86–1.92)	0.230
Multivariable HRs (95% CI)	Ref	0.83 (0.57–1.22)	0.98 (0.64–1.48)	0.599
**Uterine cancer**				
Cases (n = 173)	158	9	6	
Univariable HRs (95% CI)	Ref	0.93 (0.47–1.81)	0.86 (0.38–1.95)	0.681
Multivariable HRs (95% CI)	Ref	0.79 (0.40–1.55)	0.79 (0.34–1.81)	0.420
**Prostate cancer**				
Cases (n = 951)	885	41	25	
Univariable HRs (95% CI)	Ref	0.75 (0.55–1.03)	0.64 (0.43–0.95)	0.006
Multivariable HRs (95% CI)	Ref	1.05 (0.77–1.44)	1.07 (0.72–1.61)	0.654
**Renal cancer**				
Cases (n = 130)	119	6	5	
Univariable HRs (95% CI)	Ref	0.82 (0.36–1.86)	0.95 (0.39–2.33)	0.752
Multivariable HRs (95% CI)	Ref	0.77 (0.34–1.76)	0.86 (0.34–2.13)	0.569

Multivariable HRs were adjusted for age (continuous), sex, study area, body mass index, ethanol intake, smoking (pack-years), coffee intake, physical activity, occupation, family history of cancer, history of diabetes, and history of hypertension. Additionally, adjusted for history of chronic hepatitis or cirrhosis for liver cancer, status of menopause, and use of hormonal agents for breast and uterine cancer analysis.

In the analysis stratified by sex (men in Table 3 and women in Table 4), a different trend was observed between men and women. For lung cancer. The duration of anti-cholesterol drug use showed the tendency of the association with a decreased risk of lung cancer in men, although no significant association was observed, whereas there was a significantly increased risk of lung cancer in women (HR:1.56, 95% CI:1.03–2.35 in > 5 years group; *P* for trend = 0.032). Significant differences were observed in the HRs and *P* for trend in liver cancer in men only (HR:0.20, 95% CI:0.05–0.82 in > 5 years group; *P* for trend = 0.004).

**Table 3:** Hazard ratios for major cancer types according to anti-cholesterol drug use categories in men.

	None	< 5 years	> 5 years	*P* for trend	*P* for interaction for sex
All cancers					
Cases (n = 5,387)	5,039	215	133		
Univariable HRs (95% CI)	Ref	1.07 (0.94–1.23)	1.08 (0.91–1.28)	0.207	0.136
Multivariable HRs (95%CI)	Ref	0.95 (0.83–1.09)	0.94 (0.79–1.12)	0.364	0.506
Esophageal cancer					
Cases (n = 226)	215	5	6		
Univariable HRs (95% CI)	Ref	0.58 (0.24–1.42)	1.14 (0.51–2.57)	0.729	0.082
Multivariable HRs (95% CI)	Ref	0.58 (0.24–1.41)	1.15 (0.50–2.60)	0.729	0.070
**Stomach cancer**					
Cases (n = 979)	919	29	31		
Univariable HRs (95% CI)	Ref	0.79 (0.55–1.15)	1.38 (0.97–1.97)	0.385	0.931
Multivariable HRs (95% CI)	Ref	0.70 (0.48–1.01)	1.16 (0.80–1.66)	0.787	0.659
**Colorectal cancer**					
Cases (n = 935)	871	41	23		
Univariable HRs (95% CI)	Ref	1.18 (0.87–1.62)	1.08 (0.71–1.63)	0.395	0.905
Multivariable HRs (95% CI)	Ref	1.07 (0.78–1.47)	0.96 (0.63–1.46)	0.896	0.959
**Liver cancer**					
Cases (n = 278)	270	6	2		
Univariable HRs (95% CI)	Ref	0.56 (0.25–1.25)	0.30 (0.08–1.21)	0.028	0.800
Multivariable HRs (95% CI)	Ref	0.47 (0.21–1.07)	0.20 (0.05–0.82)	0.004	0.751
**Biliary tract cancer**					
Cases (n = 127)	114	11	2		
Univariable HRs (95% CI)	Ref	2.43 (1.31–4.51)	0.72 (0.18–2.90)	0.264	0.053
Multivariable HRs (95% CI)	Ref	1.93 (1.03–3.61)	0.53 (0.13–2.18)	0.763	0.063
**Pancreatic cancer**					
Cases (n = 173)	161	7	5		
Univariable HRs (95% CI)	Ref	1.10 (0.51–2.34)	1.27 (0.52–3.10)	0.568	0.481
Multivariable HRs (95% CI)	Ref	0.97 (0.45–2.07)	1.12 (0.46–2.76)	0.868	0.619
**Lung cancer**					
Cases (n = 752)	711	25	16		
Univariable HRs (95% CI)	Ref	0.88 (0.59–1.32)	0.92 (0.56–1.51)	0.557	0.092
Multivariable HRs (95%CI)	Ref	0.79 (0.53–1.18)	0.82 (0.50–1.35)	0.215	0.133
**Prostate cancer**					
Cases (n = 951)	885	41	25		
Univariable HRs (95% CI)	Ref	1.17 (0.85–1.59)	1.16 (0.78–1.72)	0.274	-
Multivariable HRs (95% CI)	Ref	1.05 (0.77–1.44)	1.07 (0.72–1.61)	0.654	-
**Renal cancer**					
Cases (n = 87)	82	4	1		
Univariable HRs (95% CI)	Ref	1.23 (0.45–3.35)	0.50 (0.07–3.58)	0.702	0.413
Multivariable HRs (95% CI)	Ref	0.97 (0.35–2.67)	0.40 (0.06–2.92)	0.421	0.477

Multivariable HRs were adjusted for age (continuous), sex, study area, body mass index, ethanol intake, smoking (pack-years), coffee intake, physical activity, occupation, family history of cancer, history of diabetes, and history of hypertension. Additionally, adjusted for history of chronic hepatitis or cirrhosis for liver cancer, status of menopause, and use of hormonal agents for breast and uterine cancer analysis.

**Table 4:** Hazard ratios for major cancer types according to anti-cholesterol drug use categories in women.

	None	< 5 years	> 5 years	*P* for trend
All cancers				
Cases (n = 3,388)	2,908	244	236	
Univariable HRs (95% CI)	Ref	1.05 (0.93–1.20)	1.34 (1.18–1.54)	< 0.001
Multivariable HRs (95% CI)	Ref	0.95 (0.83–1.08)	1.11 (0.97–1.27)	0.353
Esophageal cancer				
Cases (n = 28)	24	4	0	
Univariable HRs (95% CI)	Ref	2.10 (0.73–6.04)	–	0.733
Multivariable HRs (95% CI)	Ref	2.01 (0.69–5.88)	–	0.655
Stomach cancer				
Cases (n = 482)	411	29	42	
Univariable HRs (95% CI)	Ref	0.89 (0.61–1.29)	1.70 (1.24–2.33)	0.010
Multivariable HRs (95% CI)	Ref	0.76 (0.52–1.12)	1.27 (0.91–1.76)	0.489
Colorectal cancer				
Cases (n = 709)	605	61	43	
Univariable HRs (95% CI)	Ref	1.27 (0.97–1.65)	1.18 (0.87–1.61)	0.090
Multivariable HRs (95% CI)	Ref	1.12 (0.85–1.45)	0.92 (0.67–1.26)	0.900
Liver cancer				
Cases (n = 125)	115	7	3	
Univariable HRs (95% CI)	Ref	0.76 (0.36–1.64)	0.43 (0.14–1.36)	0.114
Multivariable HRs (95% CI)	Ref	0.74 (0.34–1.60)	0.37 (0.12–1.18)	0.067
Biliary tract cancer				
Cases (n = 127)	106	8	13	
Univariable HRs (95% CI)	Ref	0.95 (0.46–1.95)	2.04 (1.14–3.62)	0.040
Multivariable HRs (95% CI)	Ref	0.74 (0.36–1.52)	1.31 (0.72–2.37)	0.633
Pancreatic cancer				
Cases (n = 162)	135	9	18	
Univariable HRs (95% CI)	Ref	0.84 (0.43–1.65)	2.21 (1.35–3.62)	0.010
Multivariable HRs (95% CI)	Ref	0.75 (0.38–1.48)	1.82 (1.09–3.04)	0.087
Lung cancer				
Cases (n = 321)	267	27	27	
Univariable HRs (95% CI)	Ref	1.27 (0.85–1.89)	1.67 (1.12–2.48)	0.007
Multivariable HRs (95%CI)	Ref	1.17 (0.78–1.74)	1.56 (1.03–2.35)	0.032
Breast cancer				
Cases (n = 491)	437	29	25	
Univariable HRs (95% CI)	Ref	0.83 (0.57–1.21)	0.95 (0.63–1.42)	0.510
Multivariable HRs (95% CI)	Ref	0.84 (0.58–1.23)	0.99 (0.65–1.50)	0.662
Uterine cancer				
Cases (n = 172)	157	9	6	
Univariable HRs (95% CI)	Ref	0.72 (0.37–1.41)	0.63 (0.28–1.43)	0.163
Multivariable HRs (95% CI)	Ref	0.79 (0.40–1.55)	0.79 (0.34–1.82)	0.424
Renal cancer				
Cases (n = 43)	37	2	4	
Univariable HRs (95% CI)	Ref	0.68 (0.16–2.82)	1.79 (0.64–5.03)	0.458
Multivariable HRs (95% CI)	Ref	0.54 (0.13–2.26)	1.05 (0.36–3.05)	0.819

Multivariable HRs were adjusted for age (continuous), sex, study area, body mass index, ethanol intake, smoking (pack-years), coffee intake, physical activity, occupation, family history of cancer, history of diabetes, and history of hypertension. Additionally, adjusted for history of chronic hepatitis or cirrhosis for liver cancer, status of menopause, and use of hormonal agents for breast and uterine cancer analysis.

## Discussion

This population-based prospective study investigated the association between the long-term use of anti-cholesterol drugs and cancer risks. Since the used anti-cholesterol drugs consist of mainly statins, the association between statins with cancer risk is assumed to be reflected in the association between anti-cholesterol drugs and cancer risk. We found in the total population that the long-term use of anti-cholesterol drugs decreased the risk to develop liver cancer, but increased the risk of pancreatic cancer.

Several reports have suggested that the mechanism of action of statins exerts a protective effect against cancers, independently from their effects on lowering cholesterol ^7^, and some studies reported statins reduces risk to develop cancers^14,15^.

Our findings that the long-term use of anti-cholesterol drugs decreased the risk of liver cancers, generally show consistency with those previous studies with statins ^16,17^. Although previous studies have reported statin use is associated with decreased risk of liver cancer, no sufficient evaluation regarding the duration of long-term statin use has been performed to date, which we investigated in this study. Various mechanisms of molecular underlying the effects of statins on liver cancer have been reported. In hepatocellular cancer cell lines, atorvastatin has been shown to block both the phosphorylation and activation of Myc which is an oncogene related closely to hepatocarcinogenesis, and inhibit tumor initiation and growth ^18^. In this study, a significant trend was demonstrated for liver cancer and this finding indicates that long-term use of anti-cholesterol drugs has a protective effect on the risk of liver cancer. On the other hand, the association between liver cancer risk and low cholesterol levels was previously reported ^19^. For example, it is possible that individuals with low cholesterol levels due to hepatitis were not prescribed anti-cholesterol drugs, and were categorized into the “none”. Because those with low cholesterol levels have a higher liver cancer risk, statin use may have an association with a decreased risk of liver cancer. Health awareness and adherence to drugs may be higher in the > 5 y group than other groups and may have impact on the association.

Several previous meta-analyses have suggested that statin use decreases the esophageal cancer ^20^, stomach cancer risk ^21^, colorectal cancer ^22^, biliary trace cancer ^23^, and prostate cancer ^24^. On the contrary, significant associations of these cancers with the long-term use of anti-cholesterol drugs were not observed in this study. The previous meta-analyses included studies in which statin use was not necessarily long-term. Therefore, it may be possible that the association would not be observed if the long-term use of statins was investigated in those studies. Furthermore, in a meta-analysis for prostate cancer, there was no significant association when the studies were limited to those in which statins were used for > 5 years ^24^.

In this study, the significant effect of the long-term use of anti-cholesterol drugs on the increased risk of pancreatic cancer was observed in the > 5 y group especially in all of the total, men, and women. A previous study reported the association between the use of statins for less than 5 years and an increased risk of pancreatic cancer ^25^ and a study using data mining also reported the statin use increased pancreatic cancer risk ^26^. However, most previous reports suggested that statins have a protective effect in decreasing pancreatic cancer risk ^27^. Our findings conflicted with these results. A previous study with an animal model has suggested that statins may have carcinogenic characteristics. In reviewing the rodent carcinogenicity of lipid-lowering drugs, it was found that all statins available in 1994 promoted cancers in rodents at concentrations which were equivalent to those for the prescription common in humans ^28^. There may be some factors, such as ethnic differences, that increase cancer risks to a greater extent.

A different trend was observed between men and women in the association between the duration of anti-cholesterol drug use and the risk of lung cancer. A significant association in > 5 y group and trend toward an increased risk was demonstrated in women, whereas a trend toward a decreasing risk was shown in men, although it was not statistically significant. A null association was suggested for the total population in the current study, which was consistent with the results of several meta-analyses ^29,30^. Interestingly, the possibility that statins may increase the lung cancer risk in women was suggested in a previous report, whereas no association was observed in men ^8^. In addition, the studies which used data from the Veterans Health Care System which represented a male-dominant population showed that statin use was not associated with the risk of lung cancer ^31,32^. Overall, statins may have opposing effects on the risk of lung cancer in men versus women. No previous studies have focused on the association between the long-term use of statins and cancer risk in men versus women. Hence, further investigations are required.

The main strength of the present study is the long-term information of anti-cholesterol drug use, whereas this study has several limitations. First, the types of anti-cholesterol drugs participants took were not specified. Several types of statins may show different association. Second, the duration of anti-cholesterol drug use was defined using a survey conducted every 5 years asking the current drug use, and it is not clear whether they actually took the drugs continuously during the specified period, even if the participants answered that they took the drugs in the consecutive questionnaires. We assumed that once started on the anti-cholesterol drug, it would be perpetual, since the standard practice is that anti-cholesterol drugs are generally continued. Third, because several information related to lifestyle of the participants could be collected only at baseline, there may have been some misclassifications in the exposure category. The lifestyle factors used for adjustment, such as smoking, may have changed after baseline data were obtained and were not reflected correctly. Lastly, although the statistical model was adjusted for several possible confounding factors, unmeasured variables and confounding factors, such as cholesterol level and socioeconomic status other than occupation, could have influenced the results.

In conclusion, this study suggested that long-term use of anti-cholesterol drugs is associated with a decreased risk of liver cancer, but an increased risk of pancreatic cancers. A different trend of association between the risk of lung cancer was observed between men and women. In the future, further investigations on each anti-cholesterol drug, especially different types of statin, should be conducted in Asian populations.

## Data Availability

For information on how to submit an application for gaining access to JPHC data and/or biospecimens, please follow the instructions at http://epi.ncc.go.jp/en/jphc/805/8155.html.
